# Time Course Transcriptomic Study Reveals the Gene Regulation During Liver Development and the Correlation With Abdominal Fat Weight in Chicken

**DOI:** 10.3389/fgene.2021.723519

**Published:** 2021-09-10

**Authors:** Siyuan Xing, Ranran Liu, Guiping Zhao, Martien A. M. Groenen, Ole Madsen, Lu Liu, Maiqing Zheng, Qiao Wang, Zhou Wu, Richard P. M. A. Crooijmans, Jie Wen

**Affiliations:** ^1^State Key Laboratory of Animal Nutrition, Key Laboratory of Animal (Poultry) Genetics Breeding and Reproduction, Ministry of Agriculture and Rural Affairs, Institute of Animal Sciences, Chinese Academy of Agricultural Sciences, Beijing, China; ^2^Animal Breeding and Genomics, Wageningen University & Research, Wageningen, Netherlands

**Keywords:** transcriptome, dynamics expression, WGCNA, ACSBG2, fat deposition

## Abstract

**Background:** The liver is the central metabolic organ of animals. In chicken, knowledge on the relationship between gene expression in the liver and fat deposition during development is still limited. A time-course transcriptomic study from the embryonic (day 12) to the egg-producing period (day 180 after hatch) was performed to profile slow-growing meat type chicken liver gene expression and to investigate its correlation with abdominal fat deposition.

**Results:** The transcriptome profiles showed a separation of the different developmental stages. In total, 13,096 genes were ubiquitously expressed at all the tested developmental stages. The analysis of differentially expressed genes between adjacent developmental stages showed that biosynthesis of unsaturated fatty acids pathway was enriched from day 21 to day 140 after hatch. The correlation between liver gene expression and the trait abdominal fat weight (AFW) was analyzed by weighted gene co-expression network analysis. The genes *MFGE8*, *HHLA1*, *CKAP2*, and *ACSBG2* were identified as hub genes in AFW positively correlated modules, which suggested important roles of these genes in the lipid metabolism in chicken liver.

**Conclusion:** Our results provided a resource of developmental transcriptome profiles in chicken liver and suggested that the gene *ACSBG2* among other detected genes can be used as a candidate gene for selecting low AFW chickens.

## Introduction

In chicken, transcriptional analysis on fat deposition were mainly aimed to understand the mechanisms of fat deposition in different depots, e.g., visceral fat (Resnyk et al., 2015) and intramuscular fat (Liu et al., 2019). The liver is the central metabolic organ, and it provides many essential endocrine and exocrine functions including fat synthesis (Zorn and Wells, 2009). In mammals, the liver provides around 70% of *de novo* synthesized fatty acid (FA), whereas in chickens, the liver provides around 90% of *de novo* synthesized FA (O’Hea and Leveille, 1969). Our knowledge of chicken liver gene expression during different developmental stages and how it regulates the lipid deposition is still limited.

In the last decades, new insights into the liver–visceral adipose axis grew rapidly (Cornide-Petronio et al., 2019). An important factor influencing the liver lipid flux is the adipose tissue (Azzu et al., 2020). In the fasted state, lipolysis is the main contributor to the increased FA turnover rate, whereas in the fed state, both the disability of adipose tissue to take up lipids and the failure of insulin to suppress lipolysis can increase the FA turnover rates (Azzu et al., 2020). In addition, the liver can also facilitate lipolysis of adipose tissue (Mandard et al., 2006).

The liver is mainly composed of hepatocytes and biliary epithelial cells which differentiated from the endoderm (Zorn and Wells, 2009). Gene expression differed in chicken liver at five embryonic stages between chickens divergently selected for abdominal fat (AF), which showed that the FA metabolism and the peroxisome proliferator-activated receptor (PPAR) signaling pathways were enriched (Na et al., 2018). Chen et al. (2019) studied the transcriptome in the chicken liver after 2 weeks of high-fat feeding and found the differentially expressed genes (DEGs) mainly enriched in the cell cycle and PPAR signaling pathways (Chen et al., 2019).

However, gene expression during liver development and its relationship with adipose deposition in chicken has been investigated only to a limited extent. Here we present the results of gene expression in chicken liver at different embryonic (from embryo day 12) until egg-production (up to day 180 after hatch) stages and find potential regulator genes for AF deposition by combining the time course, co-expression, and genomic analyses.

## Materials and Methods

### Chicken Phenotypes and Samples Collection

Yellow-feathered chicken holds nearly half of the annual chicken slaughter number in recent years in China. The Jingxing-Huang is a dwarf type breed that is widely used in the meat-type chicken industry in the north of China and is considered to be a high meat quality chicken. Compared to Cobb or Ross broilers, the marketing time for Jingxing-Huang chicken is around 90 to 120 days on account of slow growing. The Jingxing-Huang breed is also feed-efficient because of dwarfism. In this study we used Jingxing-Huang chickens from the 16th generation of an intramuscular fat-up selected line, which is a yellow-feathered slow-growing dwarfism line. The genetic background of these experimental chickens has been described in our previous studies (Zhao et al., 2006; Jiang et al., 2017). At each of the following nine developmental stages: E12 (embryonic day 12), E17, D1 (day 1 after hatching), D7, D21, D56, D98, D140, and D180, liver samples of three female chickens were collected. The chickens were reared with *ad libitum* access to feed and water. The chickens were slaughtered without fasting to avoid activation of the fasting–feeding cycle of gene expression regulation. The growth curve of body weight (BW), liver weight, and abdominal fat weight (AFW) were fitted by the Logistic model using the Origin software (version 2018). Abdominal fat percentage (AFP) was calculated by AFW/BW. Abdominal fat growth rate (AFGR) was calculated as (AFW_*later*_ – AFW_*former*_)/Time. The lower margin of the liver was collected for RNA isolation and RNA sequencing of the 27 female chickens. Oil Red O stain was used on the liver sections for developmental stages E12, E17, D1, and D21.

### RNA Sequencing and Data Quality Control

The QIAGEN RNeasy Kit was used to isolate total RNA, and genomic DNA was removed by the TIANGEN DNase KIT. The RNA concentration was assessed by Nanophotometer. RNA integrity number (RIN) was assessed by Nanodrop analysis. The RIN value of all total RNA samples was larger than 7, and RNA library construction was performed by Berry Genomics (Beijing, China). Poly-A enriched RNA samples were isolated by Dynabeads mRNA DIRECT Kit (Invitrogen). The non-stranded specific RNA libraries were sequenced on the Illumina Hiseq2500 (paired end at 125 bp). After trimming of the sequencing adaptors and low quality reads (*N* > 10% in a read) by Trimmomatic (version 0.39) with default parameters (Bolger et al., 2014), the quality of the sequencing data was assessed by FastQC (version 0.11.5) (Andrews, 2010).

### Transcriptome Profiling and Differentially Expressed Genes Detection

The transcriptome data were aligned to the chicken reference genome (GRCg6a) and annotation file (Gallus.gallus.GRCg6a.95.gtf) by STAR (version 2.5.3) (Dobin et al., 2013) and assembled with Stringtie (version 1.3.3b) (Pertea et al., 2015). Raw gene counts were performed by using a Python script provided by Stringtie with parameter l = 125 (Pertea et al., 2016). Gene expression level normalization was performed by DESeq2 (version 1.22.2) (Love et al., 2014) in R (version 3.6.1), based on the experimental design as Family + Stage. The normalized gene expression data were used for all downstream analyses (Supplementary Table 3). The list of transcription factors (TF) and transcription co-factors were extracted from AnimalTFDB (v.3.0) (Hu et al., 2018). Transcriptome principal component analysis (PCA) plots were performed by sample distances calculated by *rlog* function of DESeq2 (Love et al., 2014). Genes with the expression fold change (FC) > 1.5 or FC < 0.67 and with the Benjamini–Hochberg method (Benjamini and Hochberg, 1995) adjusted-*p* < 0.05 were considered as DEGs.

### Developmental Dynamics Genes Identification and Genes Expression Pattern

The normalized gene expression data of all libraries were used for developmental dynamics genes (DDGs) detection. The DDGs were identified by the maSigPro package (version 1.46.0) (Ana et al., 2006; Nueda et al., 2014) applying a negative binomial model for the expression distribution and using the Benjiamini and Hochberg procedure to adjust the false discovery rate. Significant genes were selected by the forward method with *r*^2^ > 0.7. Gene expression pattern analysis followed the design of a single series time course. The parameters used for gene pattern clustering: counts = TRUE, min.obs = 10, and rsq = 0.6. The k.mclust = TRUE was used to calculate the optimal clusters number.

### Weighted Gene Co-expression Network Analysis

By using all detected genes with normalized expression data across all samples, a weighted genes co-expression analysis was performed by the weighted gene co-expression network analysis (WGCNA) (version 1.41) package (Langfelder and Horvath, 2008) with minor modified parameters. The low-expressed genes were filtered by the WGCNA default parameter. By using the step-by-step topology overlap matrix (TOM) construction (soft-threshold = 8, Supplementary Figure 4B) and setting the minModuleSize to 30 for module detection. The co-expression network of a given module was filtered by edges with weight < 0.15. Finally, genes with edge numbers less than or equal to 10 were filtered out. Gene co-expression networks were performed by the Cytoscape software (version 3.6.0) (Shannon et al., 2003) with the edges provided by the WGCNA “exportNetworkToCytoscape” function. The genes with the highest Σweight were identified as hub genes. The time course impulse expression of *ACSBG2* was performed by ImpulseDE (version 3.11) (Sander et al., 2017).

### Quantitative Trait Loci Information

The chicken AFW-related quantitative trait loci (QTL) regions were collected from chicken QTL data base (release 41) (Hu et al., 2019). UCSC tool lift-over^1^ was used to transform the chicken AFW QTL regions from galGal-5.0 to GRRCg6a. The candidate gene detected in this study (*ACSBG2*) and AFW-related QTL region were visualized by Gviz (version 1.34.1) and related packages in R.

### Pathway Analysis

Kyoto Encyclopedia of Genes and Genomes (KEGG) enrichment analysis resulting in dot plots and bar plots was performed using clusterProfiler package (version 3.11.1) with p.adj < 0.05 as significant (Yu et al., 2012) and org.Gg.eg.db package (version 3.8.2) (Carlson, 2019).

### Statistical Analysis

To compare the weekly gains of AF deposition between different stages, Student’s t-test was performed by R (version 3.6.0) using the functions shapiro.test for normality test and bartlett.test to test for homogeneity of the variance. The phenotypes of AFW and abdominal fat weekly gain (AFWG) data sets which did not fit the normal distribution were compared by the rank-sum test. The Least-Significant-Difference test was performed by the agricolae package (version 1.3.1) (Mendiburu, 2017). All the significance was stated at *p* < 0.05.

## Results

### The Phenotype of Liver, BW, and AFW During Development

**FIGURE 1:**
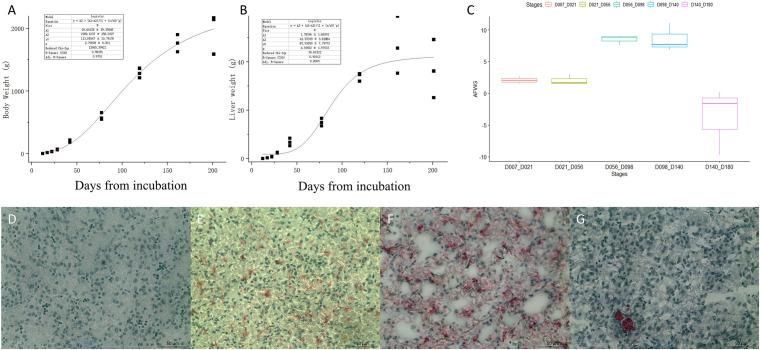
The phenotypes of body weight (BW), liver weight, and abdominal fat weekly gain (AFWG) during development. **(A)** The RNA-sequenced chicken BW fitted curve. **(B)** The sequenced chicken liver weight fitted curve. **(C)** The AFWG in different periods. **(D**–**G)** The Oil-Red-O-stained sections of the liver in E12, E17, D1, and D21, respectively. Red colors are the stained lipid or adipocytes.

Body weight, liver weight, and AFW were obtained from the 27 chickens used for RNA-seq. The fitted curves for BW and liver weight are shown in Figures 1A,B. The BW and liver weight fitted a logistic regression model. Compared with the stages from D07 to D56, the AF deposition significantly increased from D56 to D140, with more than 8 g per week (Figure 1C). The lipid analysis of the early developmental stages using the Oil Red O-stained section of the liver (Figures 1D–G) showed that there is no obvious lipid staining at E12. From E17 to the first day after hatching, the lipid started to deposit in the liver. While at D21 very limited lipid is seen in the hepatocyte, adipocytes have appeared. The phenotypic data are provided in supplementary Supplementary Table 1.

### Transcriptome Profiling During Liver Development

To observe the difference at the transcriptomic level for the nine different developmental stages, 27 RNA-seq libraries were constructed and sequenced. On average 30.61 ± 4.39 million trimmed reads were obtained per library, and the mean uniquely mapped alignment ratio was 93.78% (Supplementary Table 2). In total, 20,496 out of 24,356 genes (Gallus.gallus.GRCg6a.95.gtf) were detected as expressed (read count > 1) across the nine developmental stages of which 13,096 were ubiquitously expressed at all stages. The total number of genes expressed at each stage ranged from 15,373 in D180 to 17,222 in E12 (Table 1).

**TABLE 1:** The number of expressed, specific expressed, and switched-on/off genes.

Developmental stage	E12	E17	D1	D7	D21	D56	D98	D140	D180
No. of expressed genes	17,222	17,131	15,963	16,429	16,225	16,399	16,764	15,615	15,373
No. of specific expressed genes*	343	311	108	118	279	136	324	87	63
No. of switched-on genes^*a*^	–^*b*^	1,169	796	1,408	1,219	1,406	1,505	840	943
No. of switched-off genes^*a*^	–^*b*^	1,261	1,963	942	1,423	1,232	1,140	1,989	1,185

*^a^The list of specifically expressed gene and switched-on/off genes and the involved TFs and TF co-factors (TFCFs) in each stage are presented in Supplementary Tables 5–7. ^b^There are no switched-on/off genes at the E12.*

**FIGURE 2:**
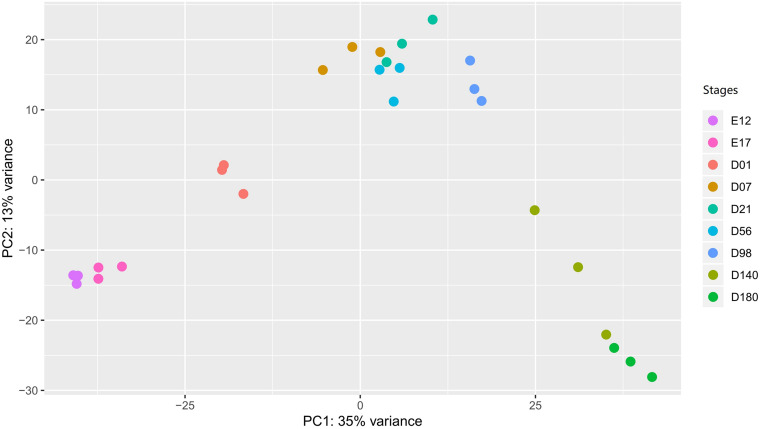
The PCA plot of liver samples in different developmental stages. In the stages, E stands for the embryonic period, and the D stands for the day after hatching. PCA, principal component analysis.

To explore whether the gene expression profiles correlated with the developmental stages, we performed a PCA plot (Figure 2). In general, the three samples of each stage clustered together. The resolution at D7 to D98 is less distinct compared to the other time points. The gene expression level of each sample is provided in Supplementary Table 3.

The number of stage-specific expressed genes varied from 63 to 343 (Table 1 and Supplementary Table 5). Stage-specific expressed genes that enriched KEGG pathways are shown in Supplementary Figure 1. The number of switched-on/off genes are presented in Table 1. Switched-on genes varied from 796 (D1) to 1,505 (D98), whereas the switched-off genes varied from 942 (D7) to 1,989 (D140). The KEGG enrichment results for the switched-on and switched-off genes are presented in Supplementary Figures 2, 3, respectively. At stage D98, the switched-on genes were enriched in alpha-linolenic acid metabolism, arachidonic acid metabolism, and ether lipid metabolism pathways. The switched-off genes at D140 are enriched in ether lipid metabolism, alpha-linolenic acid metabolism, and melanogenesis. Most of the TFs for the stage-specific expressed genes and switched-on/off genes belong to the Homeobox family (Supplementary Tables 5–7).

### Differentially Expressed Genes Between Adjacent Stages of Liver Development

**FIGURE 3:**
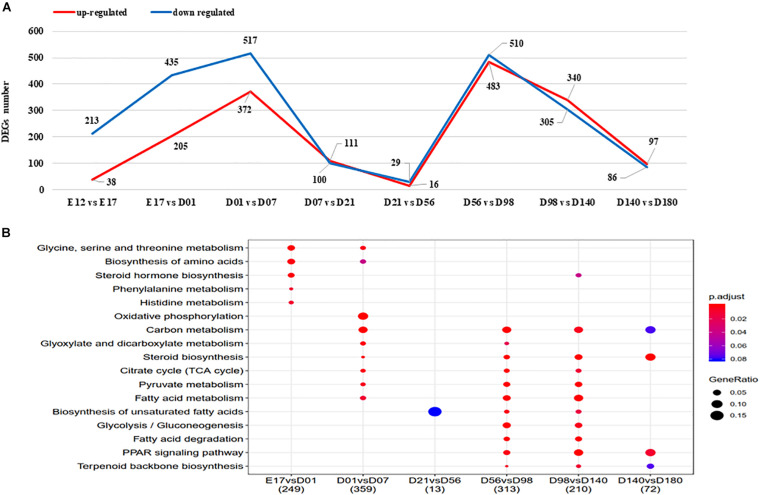
**(A)** The DEGs between adjacent stages of liver. Upregulated and downregulated gene number shown in red and blue, respectively. **(B)** The KEGG enriched pathways of DEGs between adjacent stages. DEGs, differentially expressed genes; KEGG, Kyoto Encyclopedia of Genes and Genomes.

The numbers of DEGs between the developmental stages varied from 45 (D21 vs. D56) to 4,411 (E12 vs. D180), with the detailed genes information shown in Supplementary Table 8. The number of DEGs between adjacent stages are highest for D01 vs. D07 and D56 vs. D98 (Figure 3A). KEGG pathway enrichment analysis results of the detected DEGs are presented in Figure 3B. There are no KEGG enriched pathways detected for the E12 vs. E17 nor the D7 vs. D21 comparisons. The biosynthesis of unsaturated FAs pathway was enriched for the comparison of D21 vs. D56, D56 vs. D98, and D98 vs. D140. FA degradation and PPAR signaling pathways were enriched for the comparison of D56 vs. D98 and D98 vs. D140.

### Developmental Dynamics Genes and Gene Expression Patterns in Liver

**FIGURE 4:**
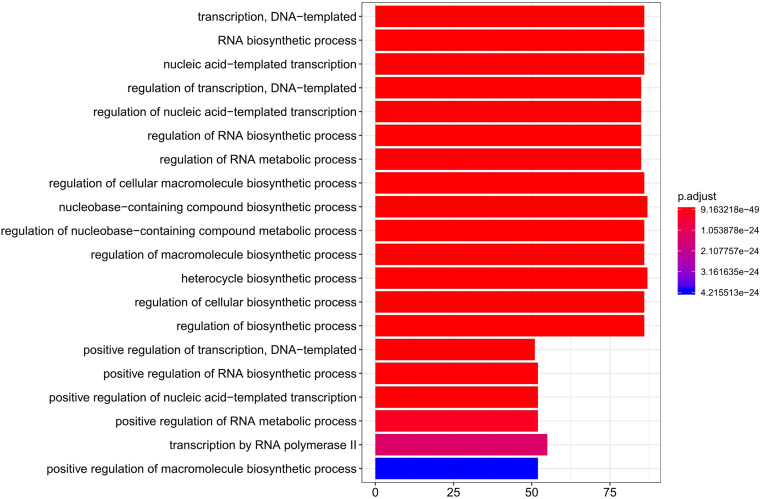
Top 20 enriched GO terms for TFs of DDGs. GO, Gene Ontology; TFs, transcription factors; DDGs, developmental dynamics genes.

To study gene expression changes during liver development, genes with significant temporal changes (DDGs) were clustered. Across all the tested stages, 8,974 genes were identified as DDGs, including 340 TFs and 261 TF co-factors (Supplementary Table 9). The top 20 enriched Gene Ontology (GO) terms for these TFs of DDGs are shown in Figure 4. Processes like DNA-templated transcription, RNA biosynthetic process, and regulation of transcription were enriched.

### Hub Genes in Liver Development and Lipid Deposition

**FIGURE 5:**
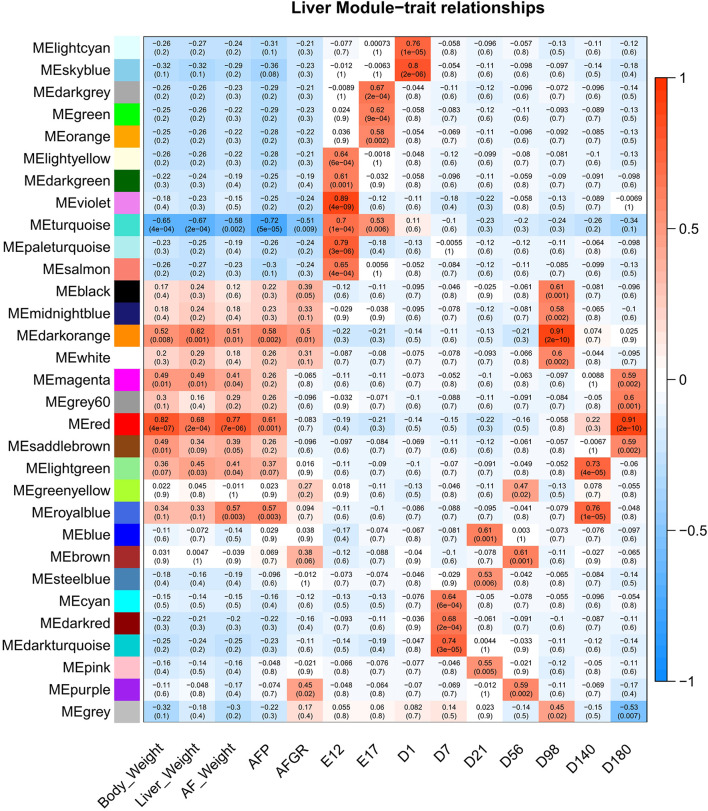
The liver expressed genes WGCNA module–trait relationship. WGCNA, weighted gene co-expression network analysis.

To detect the hub genes involved in liver growth, lipid metabolism, and AF deposition, WGCNA were performed (Supplementary Figures 4A,B). In total, 30 co-expression modules were obtained (Supplementary Figure 4C). The module–trait relationship is presented in Figure 5. The red module, involving 847 genes, is significantly positively correlated with liver weight (*p* = 2e–04), AFW (*p* = 7e–06), and AFP (*p* = 0.001). A group of 323 genes within the royal-blue module is significantly positively correlated to AFW (*p* = 0.003) and AFP (*p* = 0.003). The turquoise module is significantly negatively correlated to liver weight (*p* = 2e–04), AFW (*p* = 0.002), and AFP (*p* = 5e–05), which contains 4,852 genes in total. The turquoise module is also positively correlated to the embryonic periods E12 (*p* = 1e–04) and E17 (*p* = 0.006). The dark-orange module which includes 216 genes significantly correlated to liver weight (*p* = 0.001), AFW (*p* = 0.01), AFP (*p* = 0.002), and AFGR (*p* = 0.01). The *MFGE8* (milk fat globule-EGF 8 protein), *HHLA1* (HERV-H LTR-associating 1), *CKAP2* (cytoskeleton-associated protein 2), and *ACSBG2* (Acyl-CoA synthetase bubblegum family member 2) genes were identified as hub genes in these four modules, respectively. The pathway enrichment analyses of the genes in the four modules are presented in Table 2. The protein processing in the endoplasmic reticulum, protein export, cell cycle, DNA replication, and Fanconi anemia pathways are enriched. The co-expression network with the detected hub genes is shown in Supplementary Figures 5A–D. We found chicken QTL 24370 (chr28:1,761,021–1761061) and QTL 24371 (chr28:1,751,075–1,751,115) associated to chicken AFW that overlaps with the *ACSBG2* gene (chr28:1,746,737–1,763,012). The expression pattern of *ACSBG2* may indicate that the *ACSBG2* was impulse regulated at D98 stage (Supplementary Figure 6).

**FIGURE 6:**
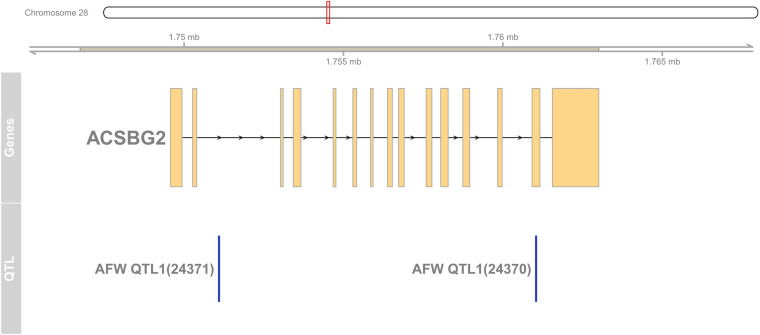
The region of *ACSBG2* covers two chicken abdominal fat weight QTLs in chromosome 28. QTL, quantitative trait loci.

**TABLE 2:** The identified hub genes, transcriptions factors (TFs), and enriched pathways.

Module	Significantly correlated to	Hub genes	Significantly enriched KEGG pathway
Red	AFW	*MFGE8*	Protein processing in endoplasmic reticulum and protein export
Royal blue	AFW	*HHLA1*	No pathway enriched
Turquoise	AFW	*CKAP2*	Cell cycle, DNA replication, and Fanconi anemia pathway
Dark orange	AFW and AFGR	*ACSBG2*	No pathway enriched

*KEGG, Kyoto Encyclopedia of Genes and Genomes; AFW, abdominal fat weight; AFGR, abdominal fat growth rate.*

## Discussion

We profiled the transcriptome during liver development from early embryonic stages to the egg-producing period in chicken. The relatively large differences of the transcriptomes during development shows a large variation of the number of expressed genes in the liver. Nevertheless, the changes in the transcriptome from D7 to D56 are relatively small. The KEGG enrichment result of temporal and ubiquitously expressed genes suggests that different biological processes are active during development. For example, D56 specific expressed genes are enriched for several FA-related pathways, e.g., linoleic acid metabolism. Furthermore, D98 switched-on genes are also enriched in linoleic acid metabolism pathway. But they are switched off in D140. This may be caused by changes in the feed composition. But it also may indicate that during D56 to D98, the linoleic acid metabolism becomes more important in liver lipid metabolism.

We identified 8,974 DDGs in this study, a number that is higher than the DDGs detected in the Red jungle fowl using both genders (Cardoso-Moreira et al., 2019). The reasons of this difference can be the different library construction methods used (single-end vs. paired-end), different reference genomes used (galGal4 vs. GRCg6a), different genders, and different time points that were used. The transcription factors of developmental dynamic genes GO enriched terms mainly focusing on the DNA-templated transcription and the regulation of RNA metabolic process. This may indicate that the TFs were regulated during developmental stages. In future studies, it would be necessary to pay more attention on the TFs that target the fat deposition-related genes.

The WGCNA is a powerful tool for complex transcriptome data sets (Langfelder and Horvath, 2008). From the modules correlating with the interest traits, we identified *MFGE8*, *HHLA1*, *CKAP2*, and *ACSBG2* as hub genes. *MFGE8*, also known as lactadherin, is a secreted glycoprotein, which can regulate hydrolysis of cytoplasmic lipid droplets in enterocytes in rats (Borisenko et al., 2004; Khalifeh-Soltani et al., 2016). *HHLA1* is a non-envelope viral sequence that is integrated into the human genome and may regulate the immune response (Balada et al., 2010). However, little is known about the function of *HHLA1* in chicken. The gene *CKAP2* regulates cell survival (Yu et al., 2015) and is the target gene of CREB, which can activate and protect mature adipocytes from apoptosis *in vitro* preadipocytes (3T3-L1) (Reusch et al., 2000). The function of the genes *MFGE8*, *HHLA1*, and *CKAP2*, in relation to fat metabolism and deposition in chicken is not completely clear. Therefore, more research is needed to determine the roles of these hub genes in liver fat metabolism and deposition in chicken, but it should be noted that some of these hub genes may be false positives caused by the positive correlation between liver weight, AFW, and BW.

Interestingly, although the corresponding correlation with AFP of the dark-orange module was lower than the red and the royal-blue modules, when we focused on the AFGR, the hub gene of dark-orange module, *ACSBG2*, shows a more potential relationship with lipid metabolism. *ACSBG2* encodes the acyl-CoA synthetase bubblegum family member 2 protein; this molecule can catalyze hexadecenoic acid to the hexadecanoyl-CoA. It is involved in the FA metabolism, FA degradation, adipocytokine signaling, PPAR signaling, and thermogenesis pathways. It also plays an important role downstream of *FAT/CD36*, which has the potential of purchasing free FA from the outside of the cell. The *ACSBG2* gene was first cloned and identified in a human in 2006 and shown to be specifically expressed in the testis and the brainstem (Pei et al., 2006). In the chicken, it is expressed in many tissues like the brain, cerebellum, heart, kidney, and ovary and highly expressed in the testis (Cardoso-Moreira et al., 2019) and liver (Supplementary Figure 6). The expression of *ACSBG2* was tested in the liver and hypothalamus tissues of fast- and slow-growing chicken by using the Affymetrix Genechip^®^ Chicken Genome array (D’Andre et al., 2013). They found two single-nucleotide polymorphisms (SNPs) in the gene significantly associated with AFW; this suggests that *ACSBG2* might be a good candidate gene for slim chicken selection. A study comparing the transcriptomes of the intestine and muscle, between divergent feed-efficient broilers, showed that *ACSBG2* influences the muscular lipid utilization and was among the highest expressed genes in muscle (Reyer et al., 2018). We, however, did not find high expression of *ACSBG2* in breast muscle at the nine different developmental stages of the same 27 slow-growing chicken used in this study (Xing et al., 2020). This difference between the studies may be caused by using different chicken breeds that differ in their growth rate.

## Conclusion

In the current study, we provided a useful gene expression data resource for chicken liver during development. The results suggest that the candidate gene *ACSBG2* among potentially other detected genes can further contribute to chicken breeding with the aim of low AFW.

## Data Availability Statement

The raw sequence data reported in this paper have been deposited in the Genome Sequence Archive (Wang et al., 2017) in Big Data Center Members (2018), Beijing Institute of Genomics (BIG), Chinese Academy of Sciences, under accession numbers CRA001334 that are publicly accessible at http://bigd.big.ac.cn/gsa.

## Ethics Statement

The animal study was reviewed and approved by the Animal Ethics Committee of Institute of Animal Sciences, Chinese Academy of Agricultural Sciences in 2016 (approved code IASCAAS-AE-02).

## Author Contributions

JW, GZ, MG, RL, RC, and SX designed the study. SX, LL, MZ, and QW performed the animal experiments and samples collection. SX and LL tested the phenotypes. SX performed data analysis and wrote the manuscript. SX, RL, RC, MG, OM, and ZW discussed the results. RL, RC, OM, MG, ZW, and JW provided valuable suggestion and comments to improve the manuscript with contributions from all other authors. All authors contributed to the article and approved the submitted version.

## Conflict of Interest

The authors declare that the research was conducted in the absence of any commercial or financial relationships that could be construed as a potential conflict of interest.

## Publisher’s Note

All claims expressed in this article are solely those of the authors and do not necessarily represent those of their affiliated organizations, or those of the publisher, the editors and the reviewers. Any product that may be evaluated in this article, or claim that may be made by its manufacturer, is not guaranteed or endorsed by the publisher.

## Footnotes

1
http://genome.ucsc.edu/cgi-bin/hgLiftOver

## Supplementary Material

Supplementary Figure 1 Liver stage-specific expressed genes enriched pathways.

Supplementary Figure 2 Liver switched-on genes enriched pathways.

Supplementary Figure 3 Liver switched-off genes enriched pathways.

Supplementary Figure 4 WGCNA processing figures. **(A)** Outliers. **(B)** Optimal soft-threshold selection. **(C)** Cluster dendrogram.

Supplementary Figure 5 Module networks. Yellow nodes stand for hub genes.

Supplementary Figure 6 *ACSBG2* impulse expressed in D98 stage.

Supplementary Table 1 Phenotypes of RNA-sequenced chickens.

Supplementary Table 2 Alignment ratio for each sequenced library.

Supplementary Table 3 Normalized gene expression matrix of each library.

Supplementary Table 4 The ubiquitously expressed gene list.

Supplementary Table 5 The stage-specific expressed gene list from E12 to D180.

Supplementary Table 6 The switch-on gene list from E17 to D180.

Supplementary Table 7 The switch-off gene list from E17 to D180.

Supplementary Table 8 The number of DEGs between each developmental stage.

Supplementary Table 9 The list of identified developmental dynamics genes (DDGs) and involved transcription factors.

## Abbreviations

AFGR: abdominal fat growth rate
AFP: abdominal fat percentage
AFW: abdominal fat weight
DDGs: developmental dynamics genes
DEGs: differentially expressed genes
QTL: quantitative trait loci
RIN: RNA integrity number
TOM: topological overlap matrix
WGCNA: weighted gene co-expression network analysis.
