# Supplementary figures and images for: Time Course Transcriptomic Study Reveals the Gene Regulation During Liver Development and the Correlation With Abdominal Fat Weight in Chicken
Source: Front Genet. 2021 Sep 10;12:723519. doi: 10.3389/fgene.2021.723519 (PMC8461244; doi:10.3389/fgene.2021.723519)

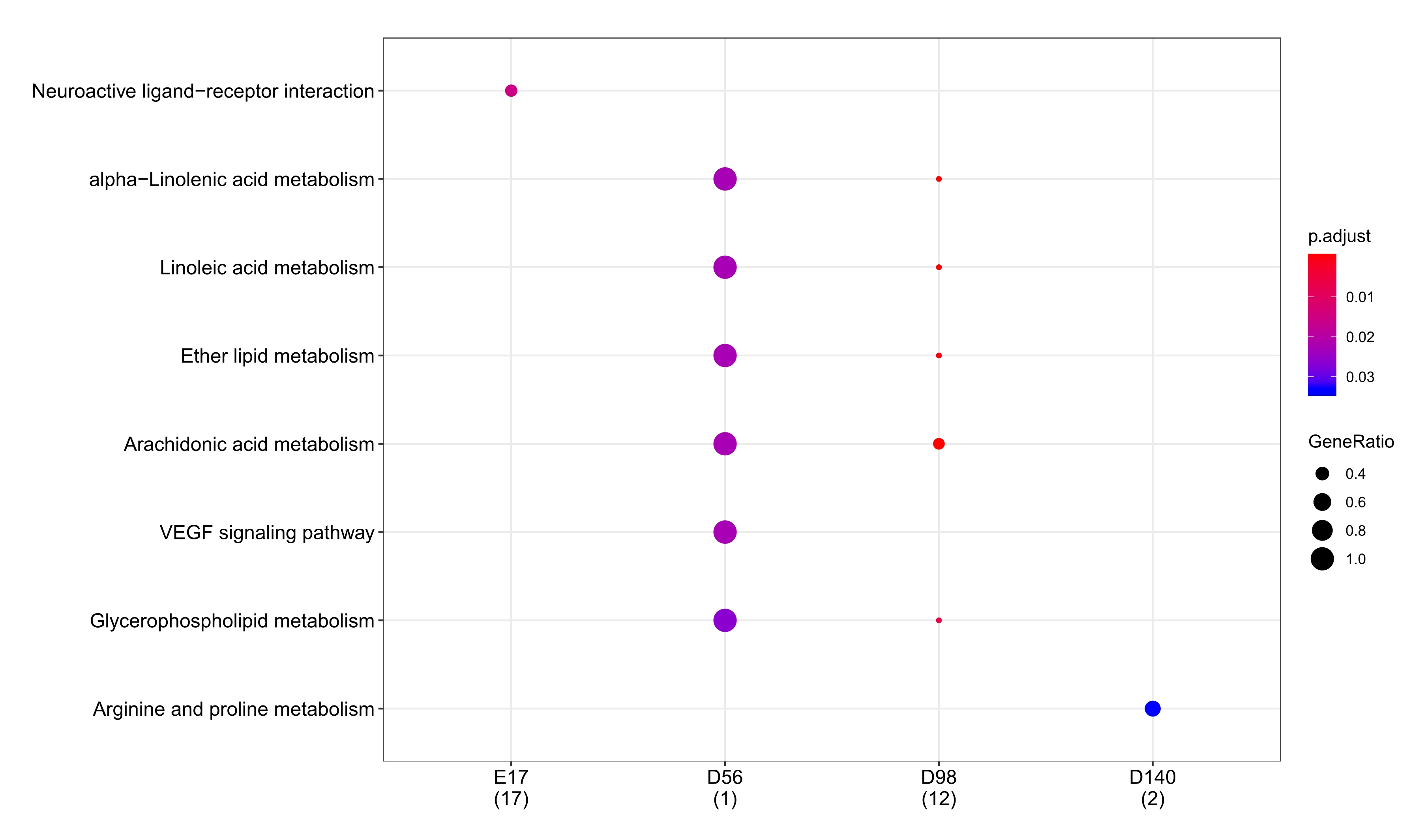

Supplement: Supplementary Figure 1 — Liver stage-specific expressed genes enriched pathways. [file Image_1.JPEG]

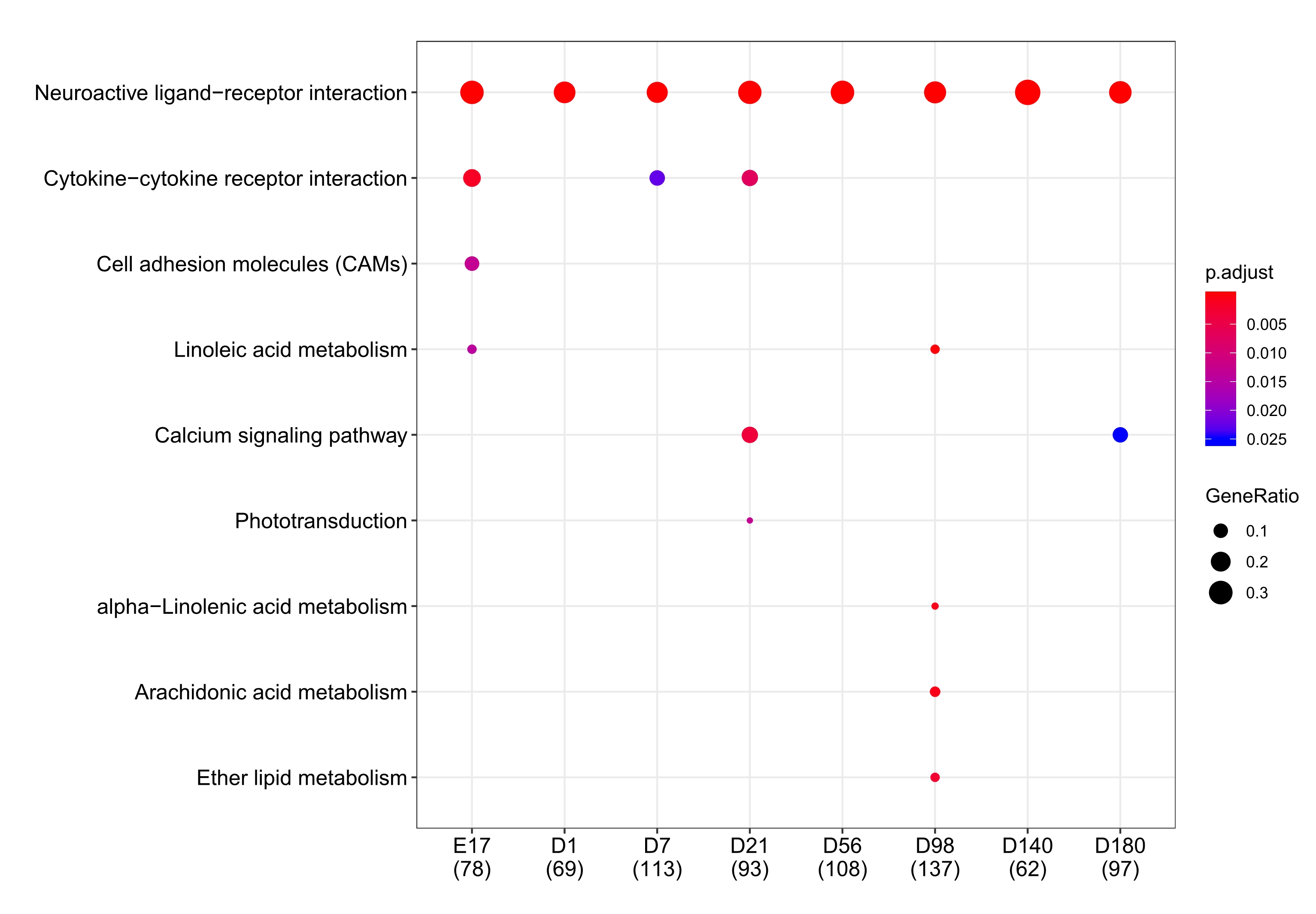

Supplement: Supplementary Figure 2 — Liver switched-on genes enriched pathways. [file Image_2.JPEG]

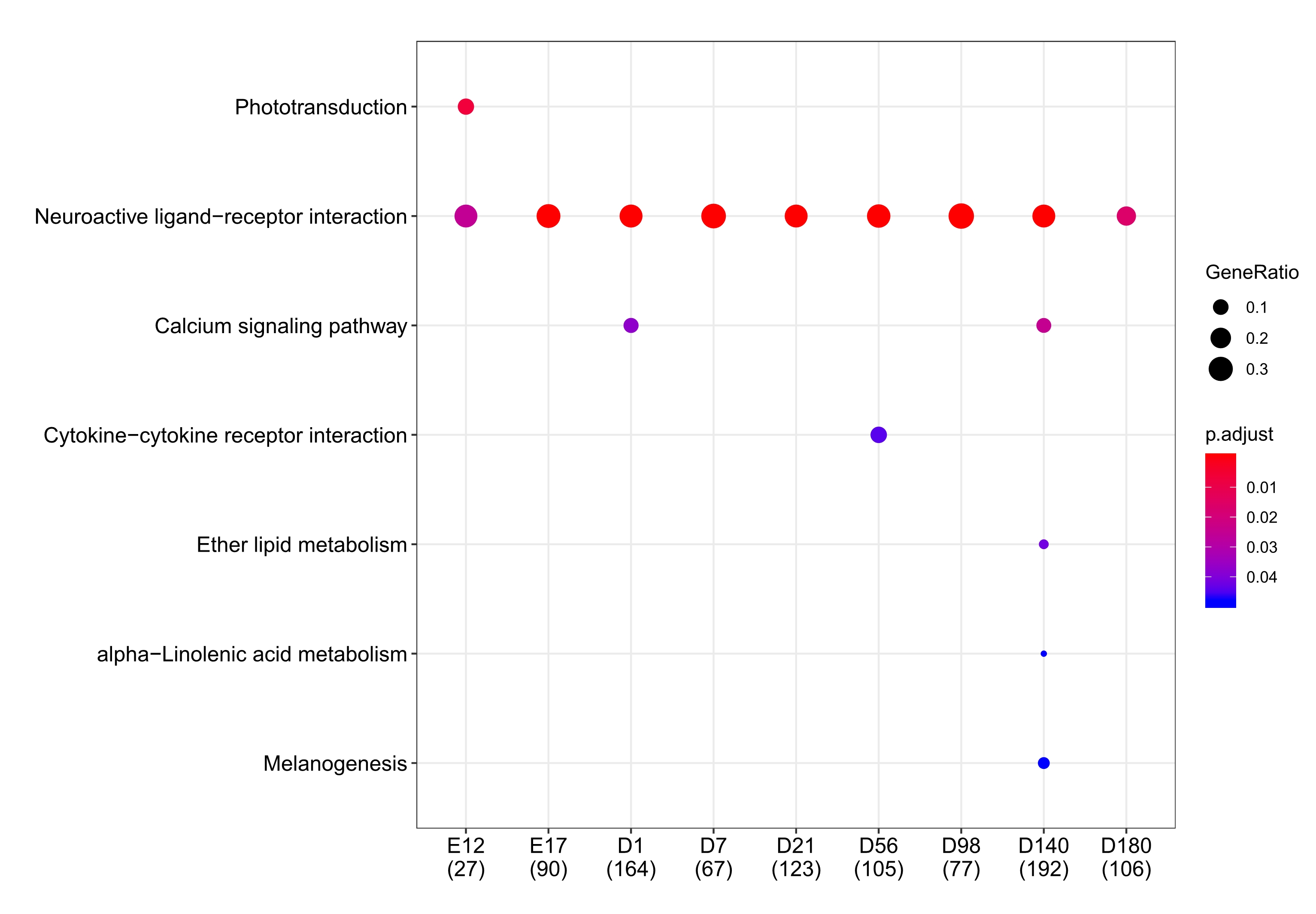

Supplement: Supplementary Figure 3 — Liver switched-off genes enriched pathways. [file Image_3.JPEG]

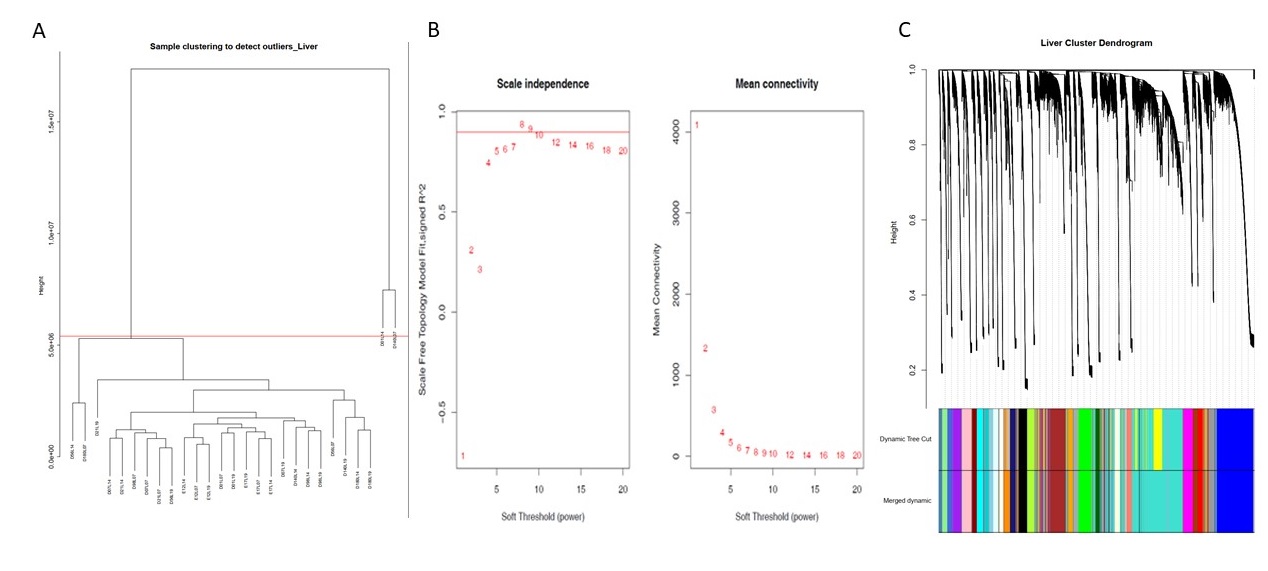

Supplement: Supplementary Figure 4 — WGCNA processing figures. (A) Outliers. (B) Optimal soft-threshold selection. (C) Cluster dendrogram. [file Image_4.JPEG]

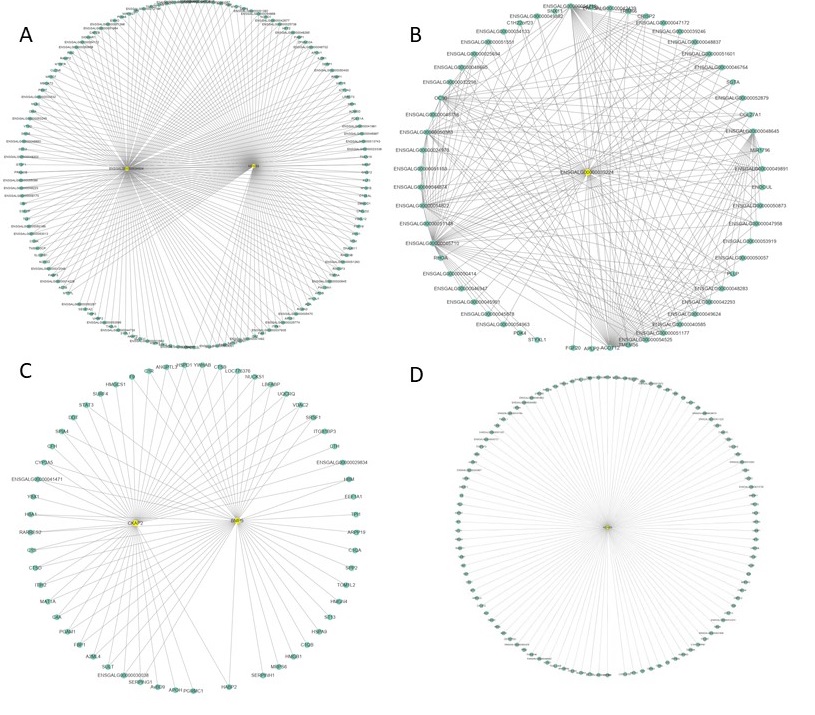

Supplement: Supplementary Figure 5 — Module networks. Yellow nodes stand for hub genes. [file Image_5.JPEG]

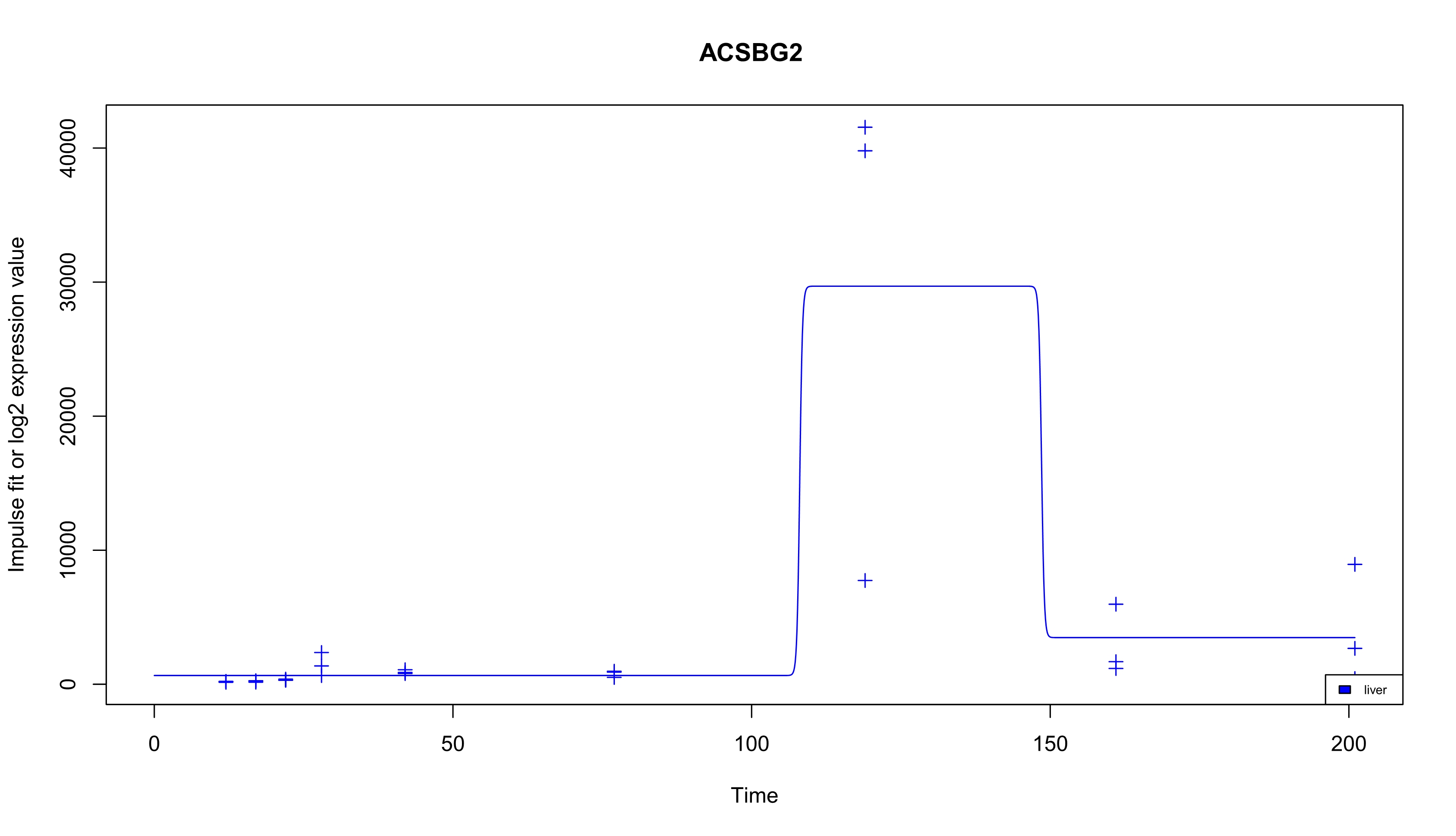

Supplement: Supplementary Figure 6 — ACSBG2 impulse expressed in D98 stage. [file Image_6.JPEG]
